# The prognostic value of lymph node ratio for thyroid cancer: a meta-analysis

**DOI:** 10.3389/fonc.2024.1333094

**Published:** 2024-02-07

**Authors:** Yue Hu, Zhiyi Wang, Lishuo Dong, Lu Zhang, Li Xiuyang

**Affiliations:** ^1^ Qi-Huang Chinese Medicine, Beijing University of Chinese Medicine, Beijing, China; ^2^ College of Traditional Chinese Medicine, Changchun University of Chinese Medicine, Changchun, China; ^3^ Department of Endocrinology, Hospital of Chengdu University of Traditional Chinese Medicine, Chengdu, China; ^4^ Guang’anmen Hospital, China Academy of Chinese Medical Sciences, Beijing, China

**Keywords:** thyroid cancer, meta-analysis, prognosis, lymph node ratio, disease-free survival

## Abstract

**Background:**

The prognostic value of lymph node ratio (LNR) has been proved in several cancers. However, the potential of LNR to be a prognostic factor for thyroid cancer has not been validated so far. This article evaluated the prognostic value of LNR for thyroid cancer through a meta-analysis.

**Methods:**

A systematic search was conducted for eligible publications that study the prognostic values of LNR for thyroid cancer in the databases of PubMed, EMBASE, Cochrane, and Web of Science up until October 24, 2023. The quality of the eligible studies was evaluated by The Newcastle-Ottawa Assessment Scale of Cohort Study. The effect measure for meta-analysis was Hazard Ratio (HR). Random effect model was used to calculate the pooled HR and 95% confidence intervals. A sensitivity analysis was applied to assess the stability of the results. Subgroup analysis and a meta-regression were performed to explore the source of heterogeneity. And a funnel plot, Begg’s and Egger’s tests were used to evaluate publication bias.

**Results:**

A total of 15,698 patients with thyroid cancer from 24 eligible studies whose quality were relatively high were included. The pooled HR was 4.74 (95% CI:3.67-6.11; P<0.05) and a moderate heterogeneity was shown (I2 = 40.8%). The results of meta-analysis were stable according to the sensitivity analysis. Similar outcome were shown in subgroup analysis that higher LNR was associated with poorer disease-free survival (DFS). Results from meta-regression indicated that a combination of 5 factors including country, treatment, type of thyroid cancer, year and whether studies control factors in design or analysis were the origin of heterogeneity.

**Conclusion:**

Higher LNR was correlated to poorer disease free survival in thyroid cancer. LNR could be a potential prognostic indicator for thyroid cancer. More effort should be made to assess the potential of LNR to be included in the risk stratification systems for thyroid cancer.

**Systematic review registration:**

https://www.crd.york.ac.uk/PROSPERO/display_record.php?RecordID=477135, identifier CRD42023477135.

## 1 Introduction

Thyroid cancer is one of the most common cancers worldwide according to the GLOBOCAN 2020 database of cancer incidence and mortality by the World Health Organization (1). In the past decades, the incidence of thyroid cancer has been increased globally (2–4). And by the year 2030, thyroid cancer will be likely to become the second most common cancer in female and the ninth most common cancer in male (1, 5–7). The three broad histological categories of thyroid cancer are differentiated thyroid cancer, medullary thyroid cancer and anaplastic thyroid cancer, of which papillary thyroid cancer, a subtype of differentiated thyroid cancer, accounts for nearly 85% of patients (8–10). The treatment for thyroid cancer is diverse and systematic, including minimally invasive interventions, surgery, radioactive iodine, oral multi-targeted tyrosine kinase inhibitors and so on (10–14). Although advances in treatment have been made in recent years, surgery remains to be the major and initial treatment in patients with differentiated and medullary thyroid cancer (9, 10).

The survival rate of thyroid cancer tend to be relatively high comparing with other types of cancers. According to the data from Surveillance, Epidemiology, and End Results(SEER) database, the 5-year relative survival of patients with thyroid cancer was 98.5% from 2013 to 2019 (15). Despite a high survival rate, postoperative recurrence is an emerging problem during thyroid cancer treatment[9]. Study results shows that 5%-20% of patients with differentiated thyroid cancer suffers from local or regional recurrence, and about 10%-15% of patients develop distant metastases (16–19). Thus, exploring reliable predictors for recurrence of thyroid cancer becomes an emerging issue.

Cumulative evidence suggests that lymph node status is a key prognostic factor in thyroid cancer (20–22). Currently, 2015 American Thyroid Association (ATA) Management Guidelines has recommended the number of involved lymph nodes to be a prognostic factor of thyroid cancer (9). However, studies have already shown that the number of metastasis lymph nodes is likely to be affected by surgical factors especially the number of lymph nodes dissected since inadequate lymph node dissection, which sometimes happens in surgery, may possibly make the number of metastasis lymph nodes lower than actual amount (23, 24). A study have even mentioned that the small number of lymph nodes removed increases the risk of recurrence in thyroid cancer (25). Therefore, the prognostic accuracy of the number of lymph nodes examined remains to be imperfect (25, 26).

Lymph node ratio (LNR) is an indicator defined as the number of metastatic lymph nodes divided by the number of lymph nodes checked. Differing from the number of metastasis lymph nodes, LNR takes the influence of both number of metastasis lymph nodes and number of lymph nodes examined into account. Thus, it is regarded as a prognostic factor in several cancers including gastric, breast, and colorectal cancers (27–29). Nevertheless, the prognostic value of LNR for thyroid cancer still remains to be controversial. Conflicting results may arise from differences in study design and sample size. Although systematic review has reported the prognostic significance of LNR for thyroid cancer, there has been no formal meta-analysis so far (30). Thus, we conducted the first comprehensive meta-analysis based on Hazard Ratios to investigate the prognostic value of LNR for thyroid cancer, and attempt to discuss the best cut-off value of LNR.

## 2 Materials and methods

### 2.1 Study selection

Inclusion criteria were defined as follows: (1)study design: retrospective and prospective cohort study reported the prognostic value of LNR for thyroid cancer (2)study content: the relationship between LNR and disease-free survival (DFS) (3)participants: patients with thyroid cancer who were standardized diagnosed and underwent thyroid surgery (4) outcome: disease-free survival (DFS) and other necessary survival data including Hazard ratios (HRs) and 95% confidence intervals (CIs) which could be extracted from the original literature directly or indirectly.

The exclusion criteria were as follows: (1) case reports, reviews, letters, conference records (2) studies conducted on animals (3) studies without necessary survival data for statistical analysis (4) studies which were based on non-original data (5) studies analyzed data from the same population.

Eligibility assessment was performed in a blinded standardized manner by 2 independent reviewers, and disagreement between reviewers were solved by consensus.

### 2.2 Search strategy

A systematic search was performed using databases including PubMed, Embase, Cochrane, and Web of Science in order to identify relevant studies from inception to October 24, 2023. Relevant medical subject heading terms, key words or word variants for “thyroid cancer” and “Lymph node ratio” were used as search items and a detailed search strategy was shown in **Table 1**. The guidelines for Preferred Reporting Items for Systemic Reviews and Meta-Analyses(PRISMA) were followed throughout the whole study.

**Table 1:** Search strategy in PubMed.

#	Term
#1	“ratio”[Title/Abstract] OR “density”[Title/Abstract]
#2	“node”[Title/Abstract] OR “nodal”[Title/Abstract]
#3	“thyroid neoplasms”[Title/Abstract] OR “thyroid cancer”[Title/Abstract] OR “thyroid carcinoma”[Title/Abstract] OR “carcinoma of thyroid”[Title/Abstract] OR “thyroid neoplasms”[MeSH Terms]
#4	#1 AND #2 AND #3

### 2.3 Data extraction

Data extracted from eligible studies consisted of basic information including first author name, country where the study was conducted and year of publication, population-specific information including number of patients, age, gender, and study-specific details including duration of follow-up, treatment for patients, tumor size, TN staging of cancer, number of checked nodes, number of positive nodes, cut-off value of LNR estimated and selected using Receiver Operating Characteristic curve based on the data from each study by investigators, HRs and 95% CIs. Two independent reviewers extracted data using a standardized data extraction form, and any discrepancy between the reviewers was resolved by consensus.

### 2.4 Quality assessment

The Newcastle-Ottawa Assessment Scale of Cohort Study was applied to the quality assessment of eligible studies. This tool evaluates the quality of studies according to 9 items across 3 dimensions: selection, comparability and outcome. A study can be awarded a maximum of one score for each numbered item within the selection and outcome categories and a maximum of two scores can be given for comparability category. 2 reviewers assessed the quality of included studies independently, and any discrepancy between the reviewers was resolved by consensus.

### 2.5 Data synthesis and statistical analysis

This meta-analysis was performed using pooled HRs and 95% CIs to evaluate the prognostic value of LNR for thyroid cancer, and results were presented using forest plots. Missing hazard ratios would be approximated using the method from Guyot et al. if Kaplan Meier curves were provided (31). A random-effects model was applied rather than a fixed-effects model to determine the robustness of the results. Heterogeneity between studies was calculated using I2 statistics which indicated the percentage of heterogeneity that is beyond chance (I2 < 25%, low heterogeneity; I2 = 25-50%, moderate heterogeneity; and I2 > 50%, strong heterogeneity) (32). A sensitivity analysis was conducted to assess the stability and robustness of the results by omitting every individual study and calculating new HRs. Moreover, we performed subgroup analysis on the basis of histological types of thyroid cancer, treatment, location, ranges of the cut-off value of LNR, whether studies control factors in design or analysis to ensure the comparability of cohorts and whether cases with extra thyroid extension (ETE) were excluded. And a meta-regression which is a joint test for all covariates was conducted to assess the potential impact of multiple factors on heterogeneity. Finally, we used a funnel plot, Begg’s test and Egger’s test to evaluate the publication bias of included studies.

All statistic analysis were performed using STATA 11.0 software. And results were considered significant statistically if the p value was less than 0.05. The protocol of this systematic review was registered in the International Prospective Register of Systematic Reviews (PROSPERO) system (ID: 42023477135).

## 3 Results

### 3.1 Study selection, characteristics, and quality assessment

**Figure 1:**
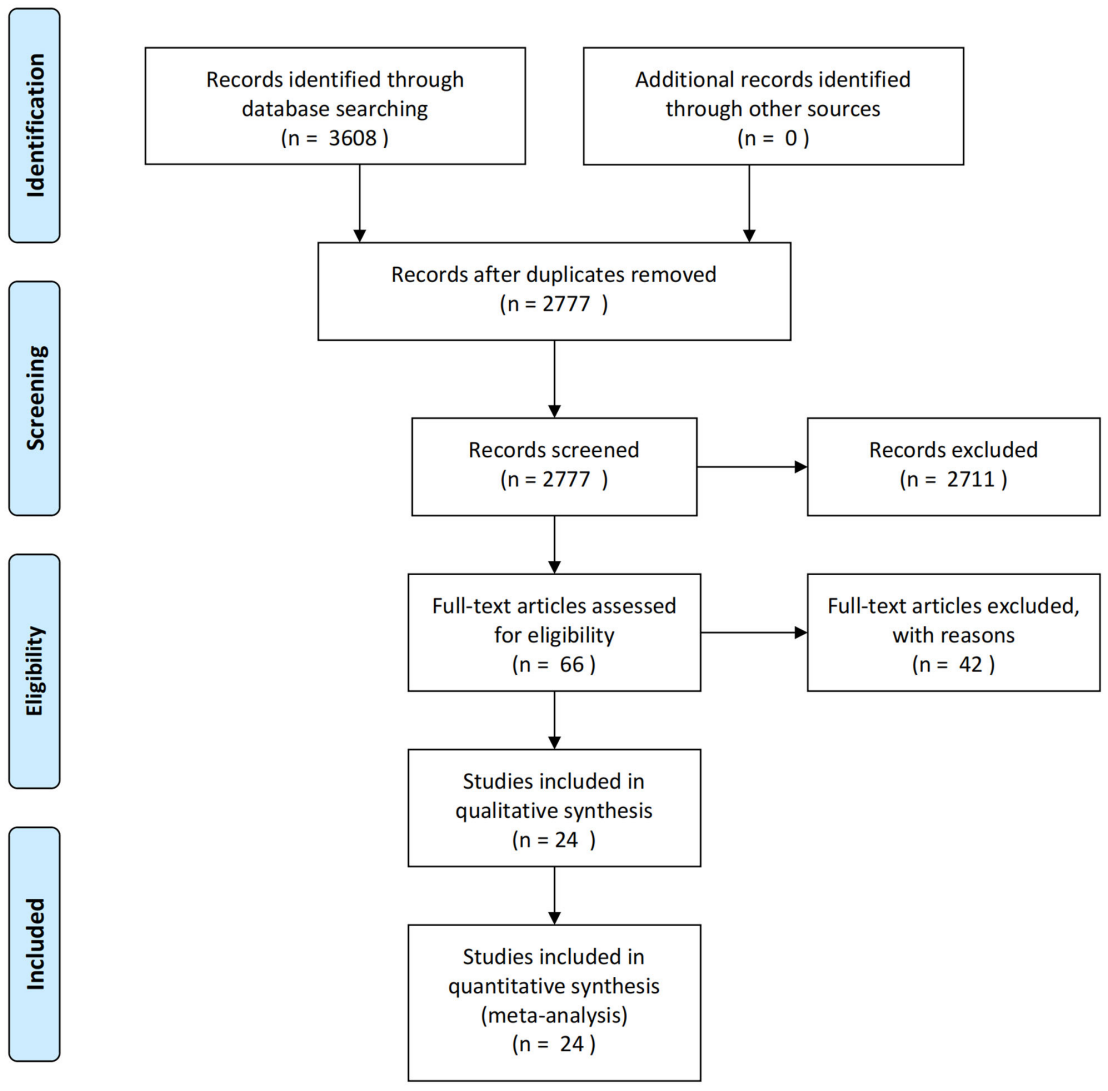
Flow diagram of included studies.

3608 articles were yielded by database searching. After reduplicates removed, 2777 articles remained for records screening. Eventually, 24 eligible articles were included after screening full texts of 66 articles according to the inclusion and exclusion criteria. And a flow diagram for study selection is shown in **Figure 1**.

All eligible studies were retrospective cohort studies, with 13 studies reported data from Korea, 4 studies from China, 2 from the United States, 2 from Israel, and 1 from Netherlands. Among these studies, a total of 15,698 patients were included, and the median age of patients ranged from 40 to 55 years old. All patients underwent radical surgery for thyroid cancer, and available median follow-up time of studies ranged from 43 to 127.24 months. The cut-off values of LNR in included studies were diverse, and only 3 studies reported 0.3 as a common cut-off values of LNR. Main characteristics of included studies were summarized in **Table 2**.

**Table 2:** Main characteristics of the included literature.

Author	Year	Country	Sample Size	Median Follow-Up Time (Months)	Treatment	Age (Years)	Male/Female	LNR	Factors Control
Weijing Hao	2023	China	160	51	surgery	52 (14-73)	70/90	0.24	Yes
Pengfei Xu	2023	China	101	50	surgery	45.1 (15-74)	49/52	0.2/0.3	No
Guo Fengli	2022	China	158	59.7	surgery	52 (19-74)	83/75	0.3	No
Anupam Kotwal	2020	United States	163	66	surgery	48.4 ± 18.8	78/85	NA	Yes
Tal Rozenblat	2020	Israel	107	93.2	surgery	50.3 (1.5-75)	50/57	0.15	Yes
Peng Guo	2023	China	495	NA	surgery	NA	350/145	0.295	Yes
II Ku Kang	2023	Korea	909	127.24	surgery+RAI	49.43 ± 12.6	176/733	0.29	No
Helene Lindfors	2023	Sweden	327	103	surgery	44 (10-85)	82/245	0.21	Yes
Hyesung Kim	2022	Korea	251	100.7	surgery	NA	80/171	0.32	Yes
Narin NCN.	2022	Israel	183	48	surgery+RAI	46.51 ± 17.77	69/114	NA	No
Na Lae Eun	2021	Korea	692	66.5	surgery+RAI	NA	228/464	NA	No
Jungirl Seok	2021	Korea	2409	58.8	surgery	49.2 (41.5-56.4)	407/2002	0.282	No
Schneider, DF	2013	United states	69	NA	surgery+RAI	40 (18-88)	28/41	0.7	No
Nunes, JHV	2013	Netherlands	198	36	surgery+RAI	45 (17.8-94.3)	68/130	NA	No
Min Ji Jeon	2013	Korea	292	96	surgery+RAI	44.4 (35.1–54.2)	28/264	0.4	No
In Sun Ryu	2013	Korea	295	78	surgery	45 (19–79)	67/228	0.65	No
Young Jae Ryu	2019	Korea	1082	78	surgery+RAI	46 (15-75)	213/869	0.5	No
Moran Amit	2018	US	2542	55	surgery+RAI	48 (18-97)	741/1801	0.19	No
Yul Hwangbo	2017	Korea	727	69.6	surgery+RAI	47 ± 11	NA	0.19	No
Yehree Kim	2017	Korea	1928	94	surgery+RAI	53 (15–86)	335/1573	0.5	No
Young Chan Lee	2017	Korea	211	43	surgery+RAI	55 (21-88)	57/154	0.26	No
Jong-Lyel Roh	2017	Korea	2071	96	surgery+RAI	54 (18-86)	423/1648	0.3	No
Young Woo Chang	2015	Korea	192	63	surgery+RAI	46 (16–76)	18/174	0.48	No
Chang Wook Lee	2015	Korea	136	62	surgery+RAI	51 (11–81)	40/96	0.26	No

NA, Not Applicable.

All included studies got no less than 6 scores in Newcastle-Ottawa scale and 4 studies got full score, indicating relatively high quality of eligible studies (**Table 3**).

**Table 3:** Quality of included studies.

Author	Selection (Scores)	Comparability (Scores)	Outcome (Scores)	Total (Scores)
Weijing Hao	4	1	3	8
Pengfei Xu	4	0	3	7
Guo Fengli	4	0	3	7
Anupam Kotwal	4	2	3	9
Tal Rozenblat	4	2	3	9
Peng Guo	4	2	2	8
II Ku Kang	4	0	3	7
Helene Lindfors	4	2	3	9
Hyesung Kim	4	2	3	9
Narin NCN	4	0	3	7
Na Lae Eun	4	0	3	7
Jungirl Seok	4	0	3	7
Schneider, DF	4	0	2	6
Nunes, JHV	4	0	3	7
Min Ji Jeon	4	0	3	7
In Sun Ryu	4	0	3	7
Young Jae Ryu	4	0	3	7

### 3.2 Meta-analysis

**Figure 2:**
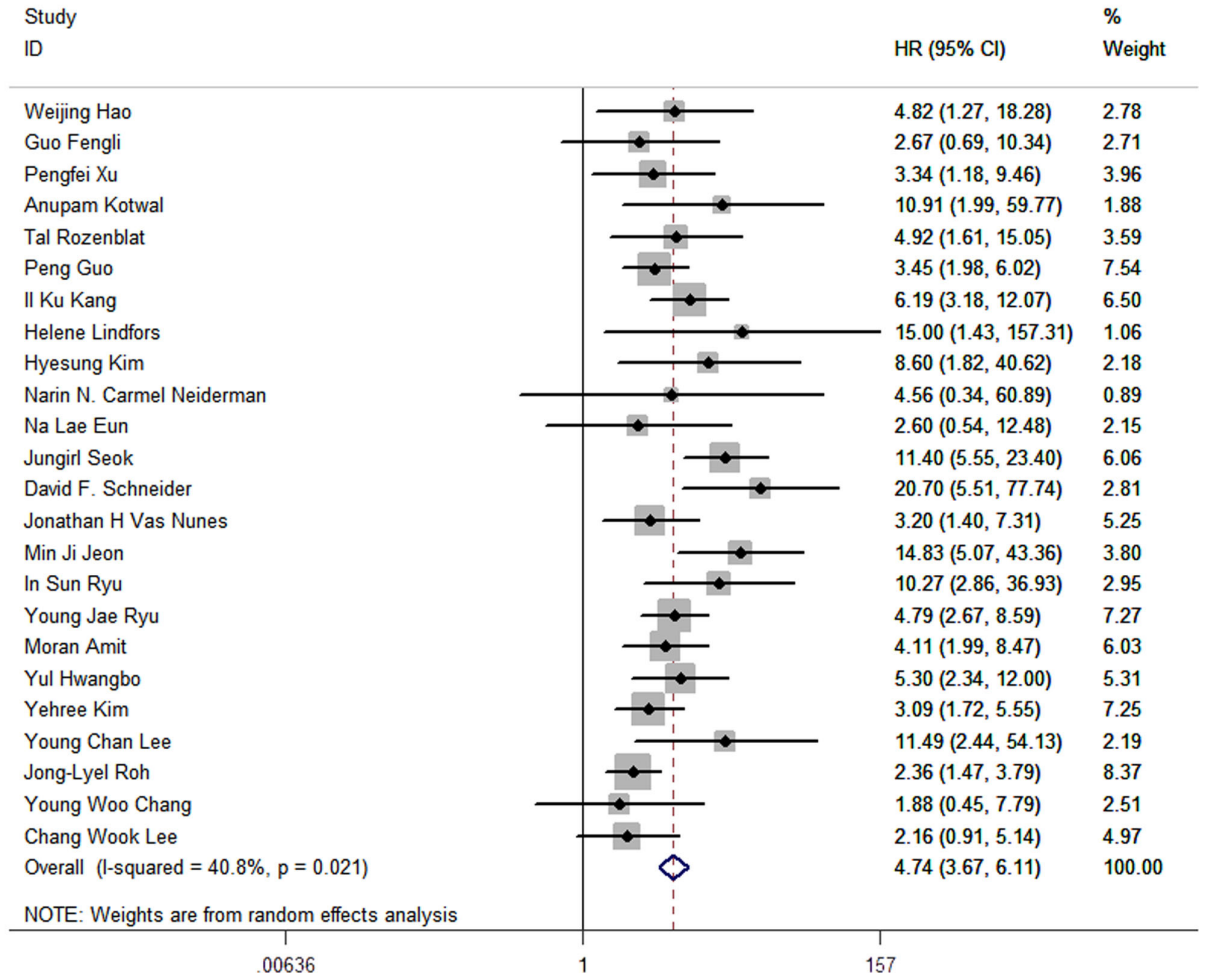
Forest plots and pooled estimates of the effect for meta-analysis of the association between LNR and disease-free survival in patients with thyroid cancer.

The results of meta-analysis of all 24 studies indicated that a higher LNR was associated with worse DFS (pooled HR: 4.74; 95% CI:3.67-6.11; P<0.05) and a moderate heterogeneity of 40.8% was reported (**Figure 2**).

### 3.3 Sensitivity analysis

**Figure 3:**
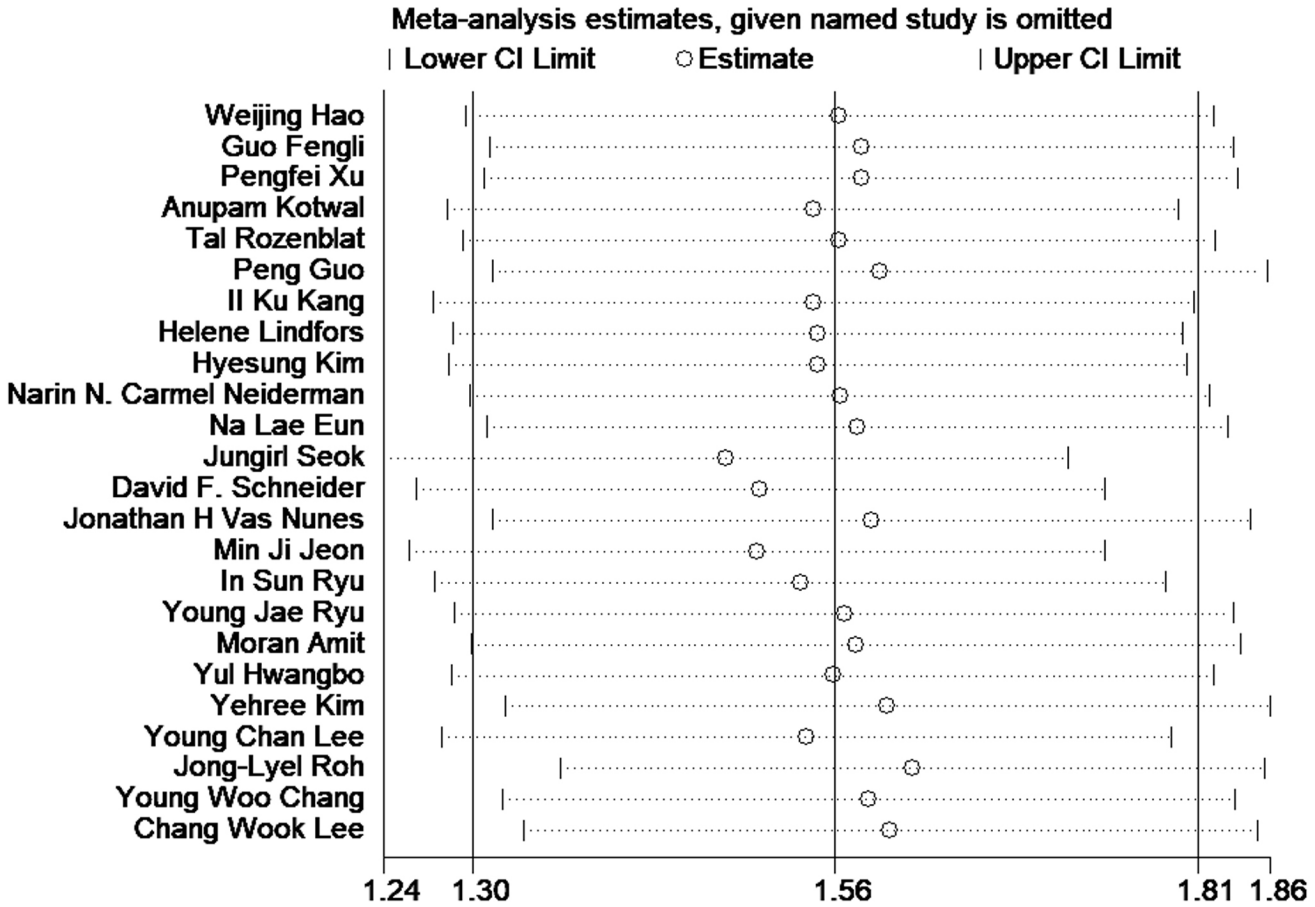
Sensitivity analysis of the association between LNR and disease-free survival in patients with thyroid cancer.

A sensitivity analysis was performed to assess the stability of the outcomes of meta-analysis. And the results intuitively showed the robust of the outcomes (**Figure 3**).

### 3.4 Subgroup analyses and meta-regression

We performed subgroup analyses based on factors including histological types of thyroid cancer, treatment, location, ranges of cut-off value of LNR and whether studies control factors in design or analysis.

The results showed that higher LNRs were correlated to poor DFS in every subgroup.

**Figure 4:**
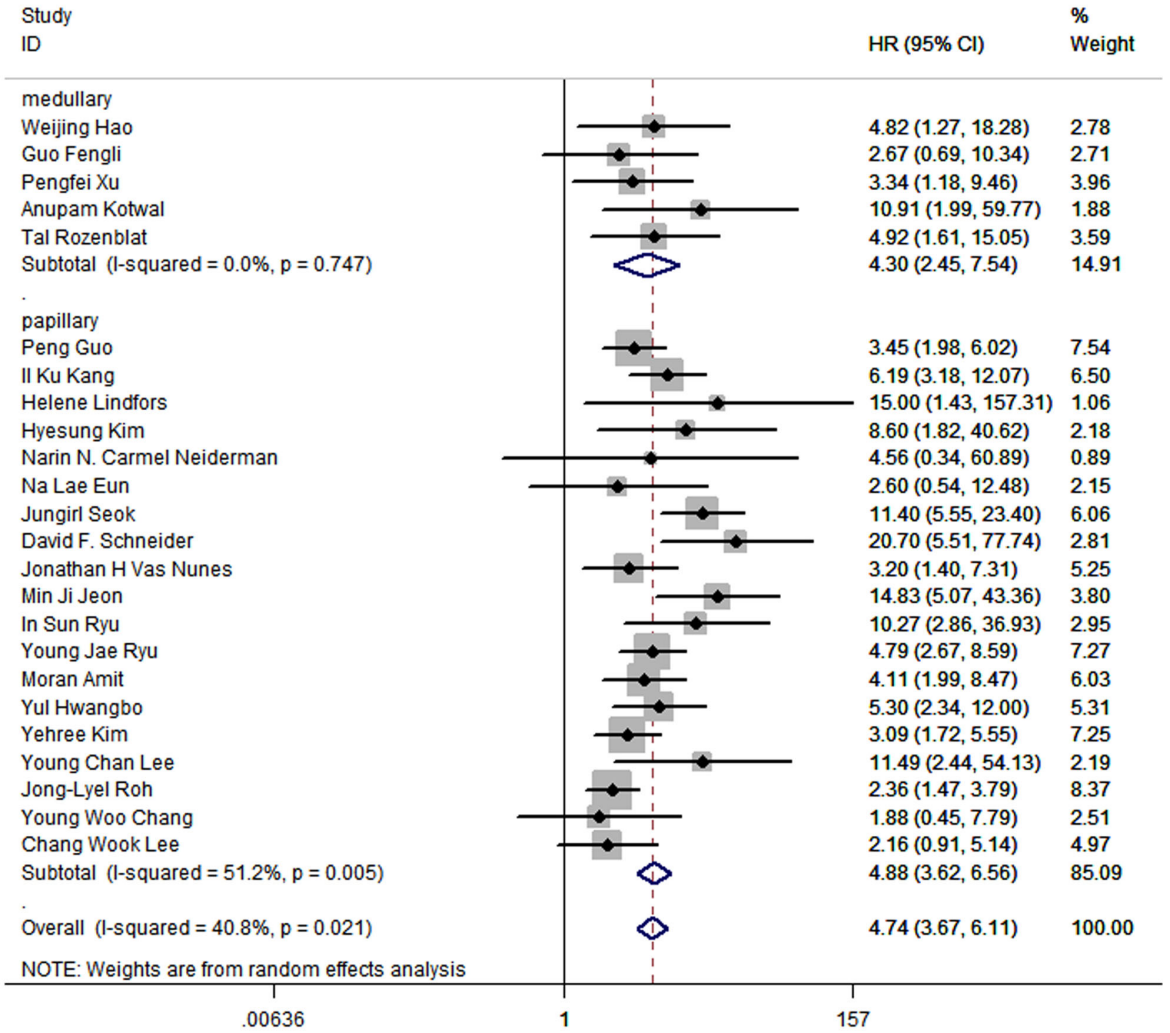
Forest plot for subgroup analysis by histological type of thyroid cancer.

In all 24 articles, 5 of them studied medullary thyroid cancer, and the other 19 studied papillary thyroid cancer. The outcomes in medullary thyroid cancer (HR:4.30; 95%CI: 2.45-7.54; P<0.05; I2 = 0.0%) were similar to the outcomes in papillary thyroid cancer (HR: 4.88, 95%CI: 3.62-6.56; P<0.05; I2 = 51.2%), while the prognostic value of LNR was slightly higher in papillary thyroid cancer (**Figure 4**).

**Figure 5:**
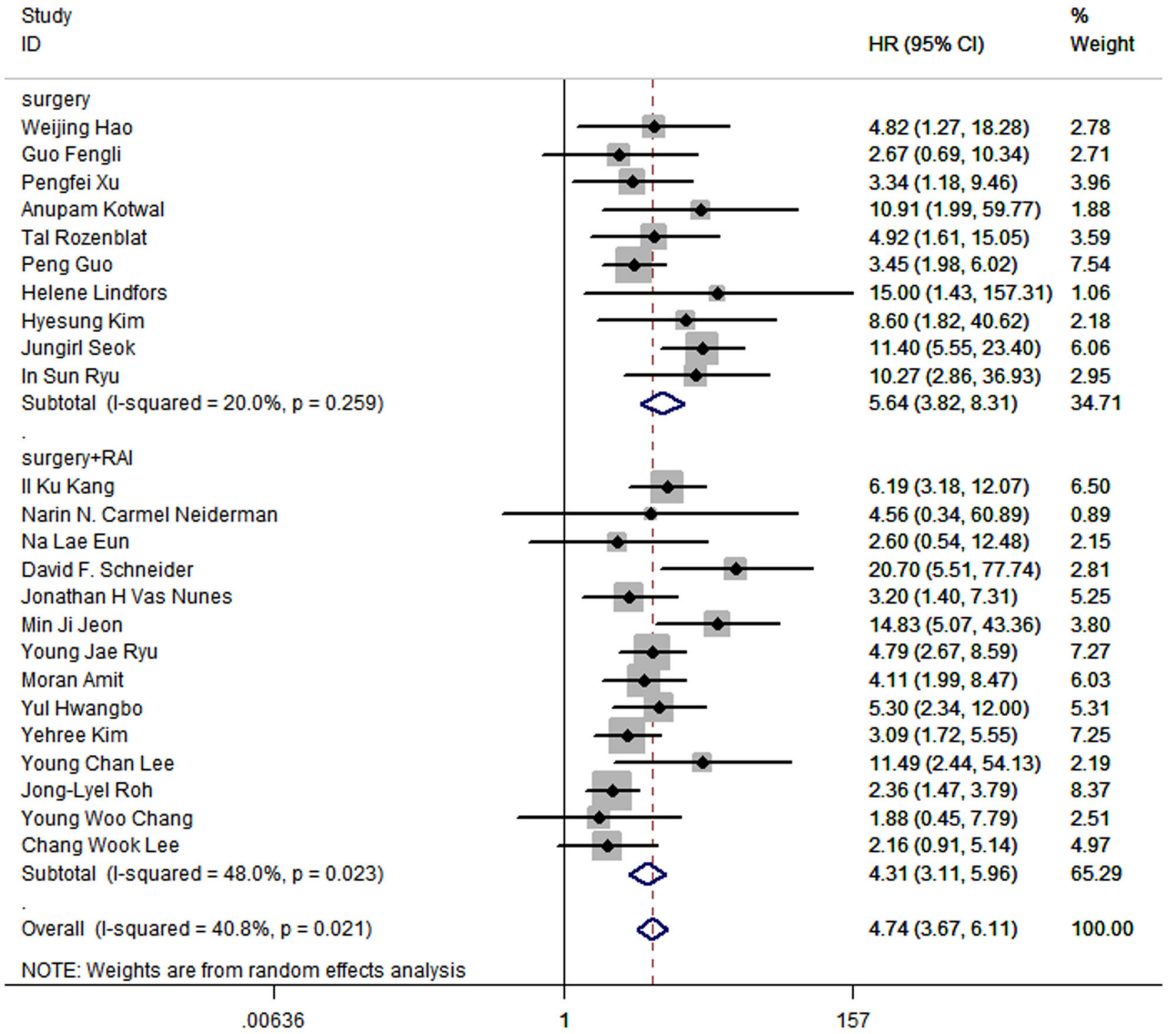
Forest plot for subgroup analysis by treatment for thyroid cancer.

In terms of treatment, 15 studies included patients who received radical surgery and RAI, while patients in other 9 studies underwent radical surgery. The pooled HR of surgery group was 5.64 (95% CI: 3.82-8.31; P<0.05; I2 = 20.0%), and the pooled HR of surgery and RAI group was 4.31 (95%CI: 3.11-5.96; P<0.05; I2 = 48.0%), which suggested that the prognostic value of LNR is significant regardless of whether RAI was used (**Figure 5**).

**Figure 6:**
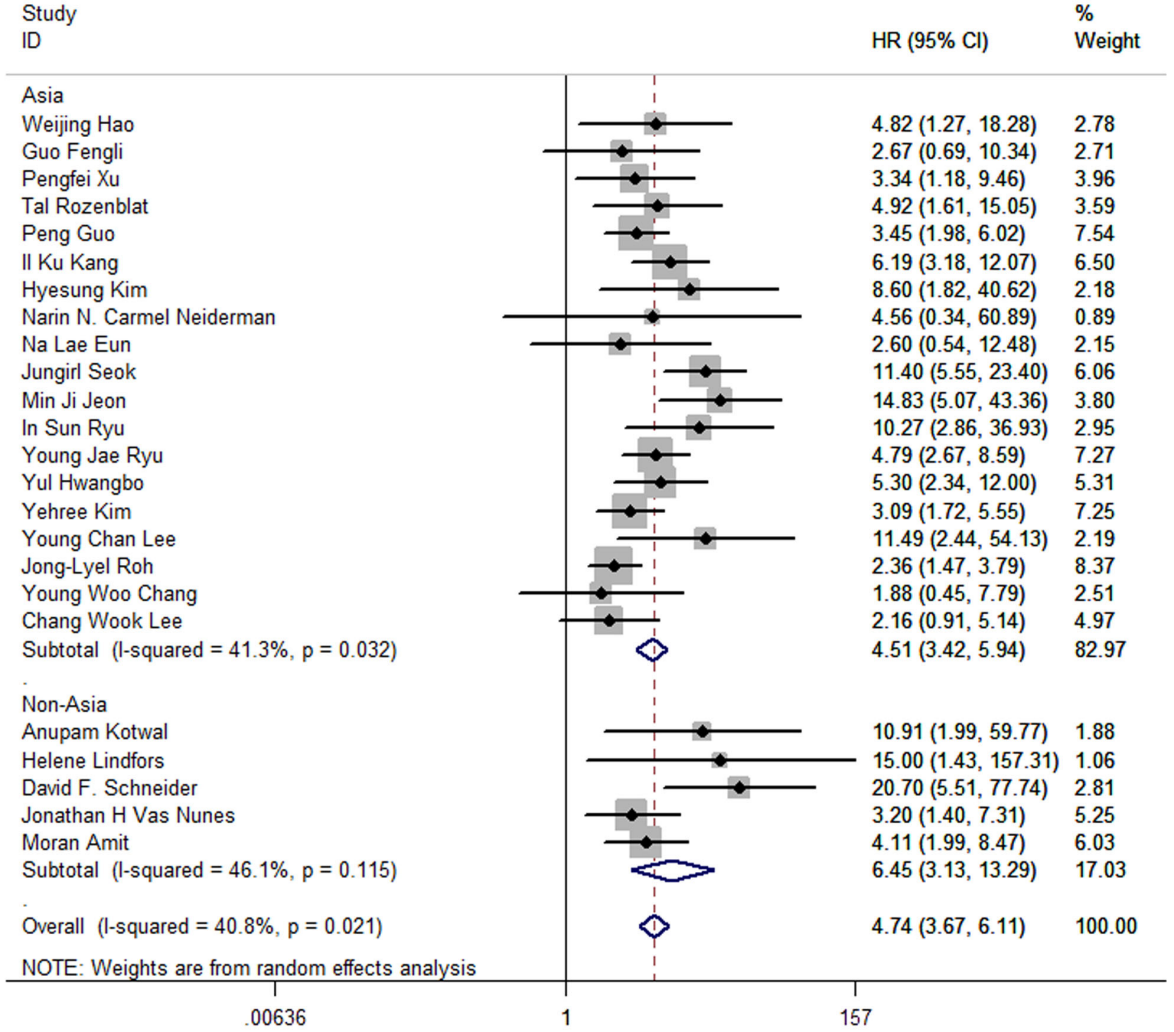
Forest plot for subgroup analysis by location of studies.

Moreover, the pooled HR of 19 Asia studies was 4.51 (95%CI: 3.42-5.94; P<0.05; I2 = 41.3%), and the pooled HR of 5 studies conducted in America and Europe was 6.45 (95%CI: 3.13-13.29; P<0.05; I2 = 46.1%) (**Figure 6**).

**Figure 7:**
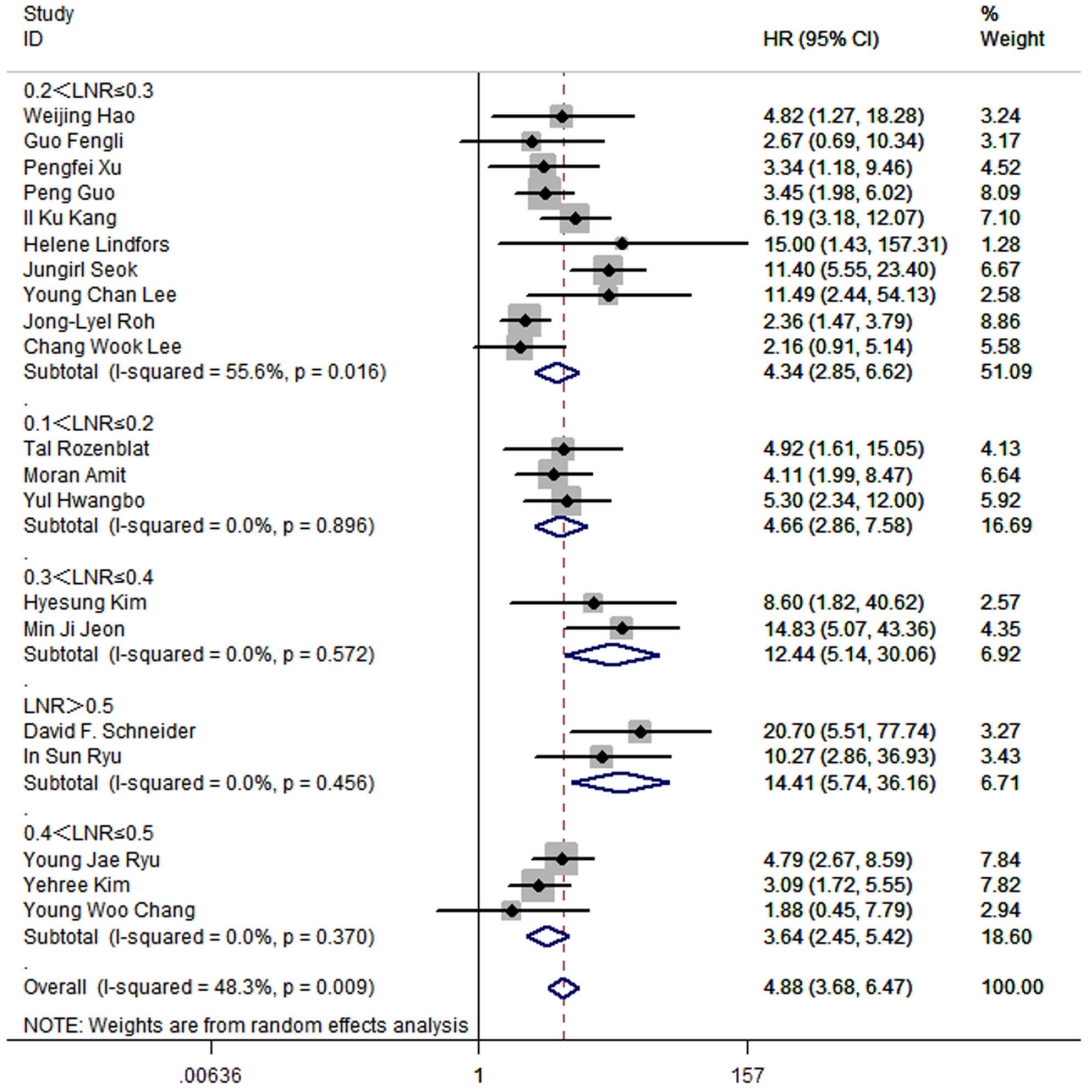
Forest plot for subgroup analysis by ranges of LNR cut-off values.

To investigate optimal range of cut-off value of LNR, studies were divided into 5 subgroups. For the 10 studies that set the cut-off value of LNR between 0.2 and 0.3, the pooled HR was 4.34 (95%CI: 2.85-6.62; P<0.05; I2 = 55.6%). For the 3 studies that set the cut-off value of LNR between 0.1 and 0.2, the pooled HR was 4.66 (95%CI: 2.86-7.58; P<0.05; I^2^ = 0.0%). 2 studies set the cut-off value between 0.3 and 0.4, and the pooled HR was 12.44 (95%CI: 5.14-30.06; P<0.05; I2 = 0.0%). 3 studies set the cut-off value between 0.4 and 0.5, and the pooled HR was 3.64 (95%CI: 2.45-5.42; P<0.05; I2 = 0.0%). 2 studies made the cut-off value of LNR greater than 0.5, and the pooled HR was 14.41 (95%CI: 5.74-36.16; P<0.05; I2 = 0.0%) (**Figure 7**).

**Figure 8:**
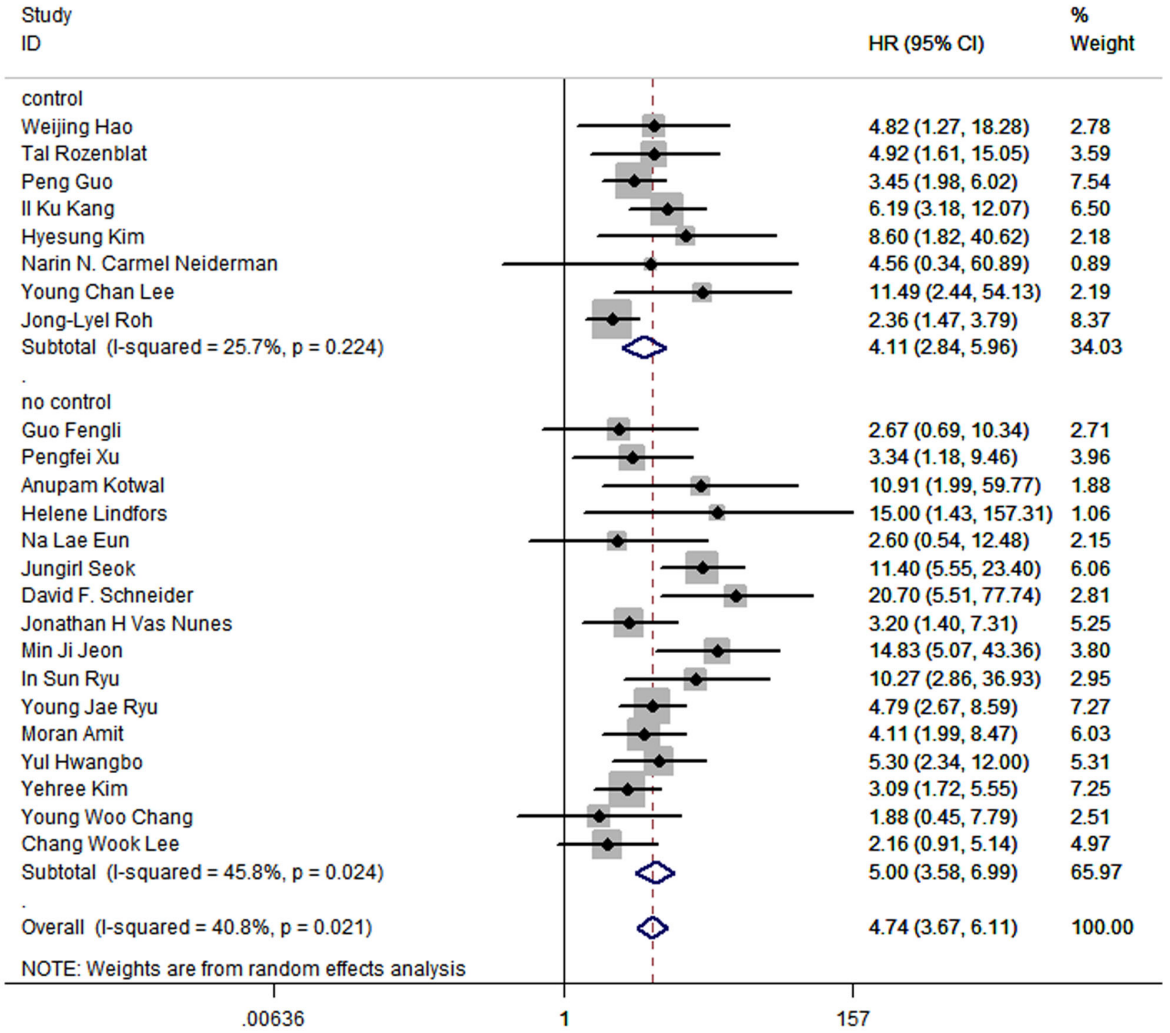
Forest plot for subgroup analysis by whether studies control factors in design or analysis to ensure the comparability of cohorts.

Furthermore, the pooled HR in subgroup of which studies control factors in design or analysis to ensure the comparability of cohorts was 4.11 (95%CI: 2.84-5.96; P<0.05; I2 = 25.7%), while the pooled HR in subgroup that did not control any factors was 5.00 (95%CI:3.58-6.99; P<0.05; I2 = 45.8%) (**Figure 8**).

**Figure 9:**
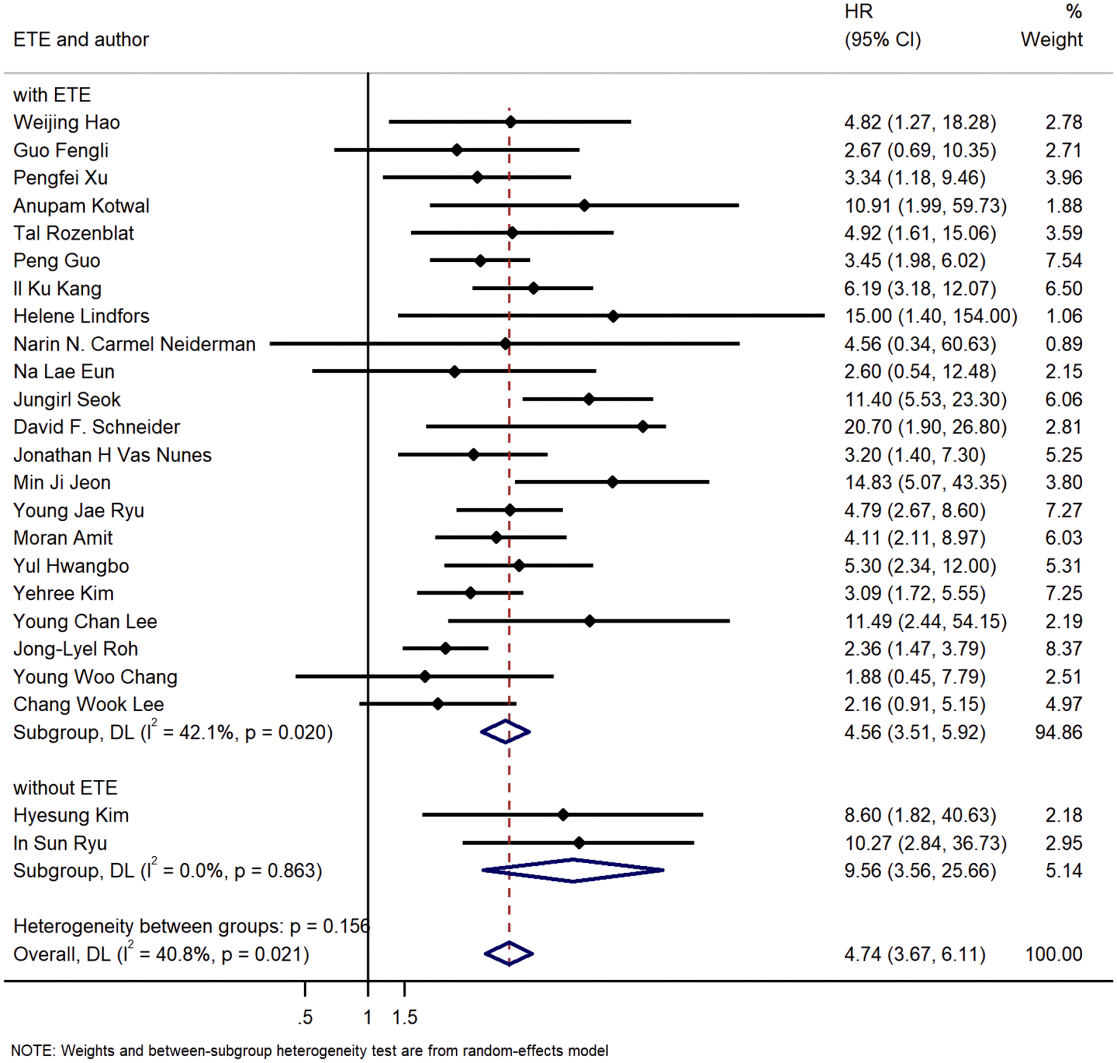
Forest plot for subgroup analysis by whether cases with extra thyroid extension were excluded.

Additionally, 22 studies included patients with ETE, while other 2 studies did not. The pooled HR of group with ETE was 4.56 (95%CI: 3.51, 5.92; P<0.05; I2 = 42.1%), and the pooled HR of group without ETE was 9.56 (95%CI: 3.56, 25.66; P<0.05; I2 = 0.0%) (**Figure 9**).

Besides, We performed a meta-regression to evaluate the influence of different factors including country, treatment, type of thyroid cancer, year and whether studies control factors in design or analysis to the pooled HR, and to clarify the origin of heterogeneity. Results suggested that the combination of these 5 factors could explain 73.01% of the variance among studies (P= 0.0444) (**Table 4**).

**Table 4:** Results of meta-regression.

Co-factor	Coefficient	95% Confidence Interval	P Value
Country	-0.32	(-0.54, -0.10)	0.006
Treatment	-0.86	(-1.54, -1.80)	0.016
Control	-0.21	(-0.72, 0.31)	0.413
Type	0.42	(-0.38, 1.23)	0.283
Year	0.03	(-0.07, 0.12)	0.549

### 3.5 Publication bias

**Figure 10:**
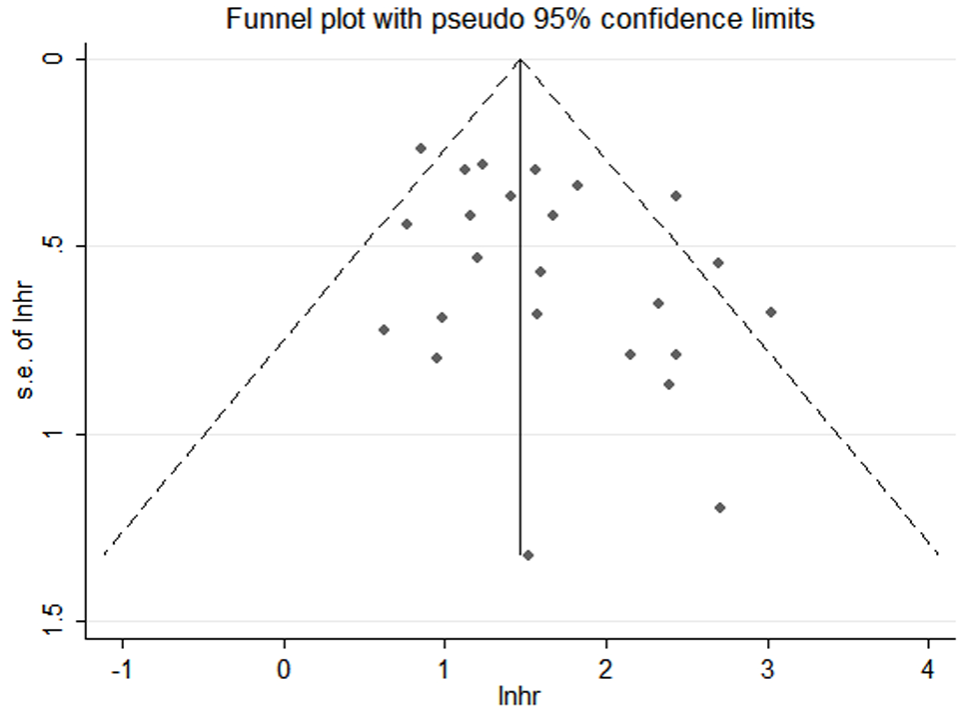
Funnel plot of included studies.

**Figure 11:**
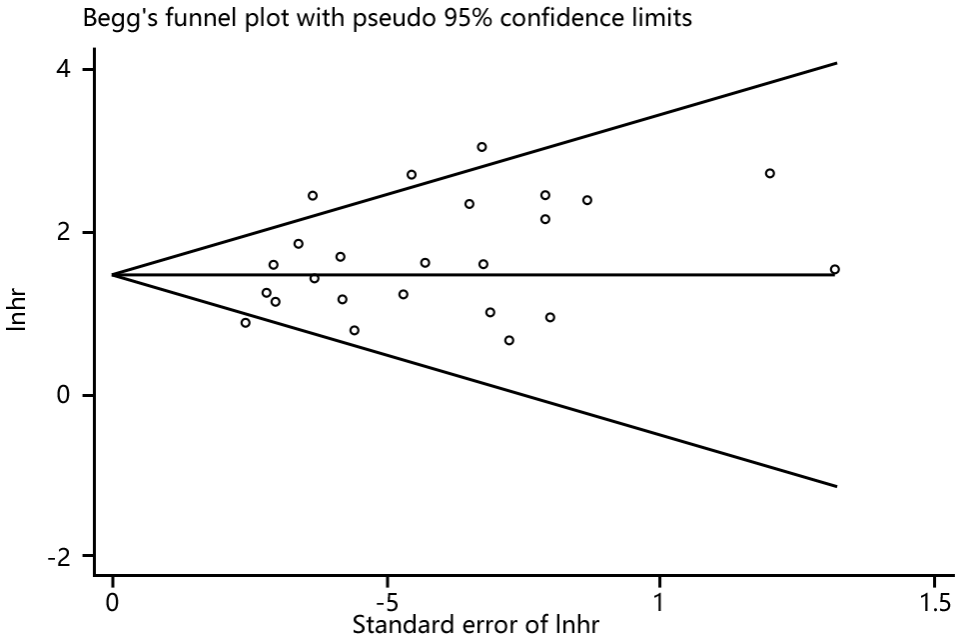
Begg’s funnel plot with pseudo 95%confidence limits.

Funnel plots, Begg’s and Egger’s tests were done to evaluate publication bias, and results showed no significant publication bias (Begg’s test: P=0.286; Egger’s test: P=0.053) in the meta-analysis (**Figures 10**, **11**).

## 4 Discussion

LNR is proved to be a significant prognostic factor for tumor recurrence and survival in multiple cancers. Several meta-analysis have suggested that high LNR is associated with low survival rates for patients with cancers (27–29, 33, 34). However, the value of LNR as prognostic variable has not been confirmed in thyroid cancer. 2015 ATA Management Guidelines has considered the number of involved lymph node to be a factor predicting recurrence, and the 2018 AJCC/TNM staging has only mentioned lymph node metastasis as the basis of thyroid cancer staging (35). It is should be noted that the number of involved lymph node metastasis are likely to be influenced by numerous factors, among which the number of lymph node examined is a major factor (25, 26). LNR, as a new indicator, takes both the number of lymph node involved and the number of checked lymph node into consideration (36). Therefore, many of studies have attempted to assess the potential of LNR to be a prognostic variable for thyroid cancer in order to provide more scientific guidance for staging,prognostic assessment, and treatment (37, 38). Unfortunately, no consensus currently exists because in addition to conflicting study results, the controversy of critical value of LNR is also a challenge on the evaluation of prognostic value of LNR for thyroid cancer.

Our study was the first formal meta-analysis to evaluate the prognostic value of LNR for thyroid cancer. In this meta-analysis,we found that high LNR was correlated with poor disease-free survival which meant LNR was a valuable potential prognostic factor for thyroid cancer. This result did not change in any type of thyroid cancer we analyzed including papillary and medullary thyroid cancer, while the prognostic efficacy was slightly higher in papillary thyroid cancer. Differences in prognostic value of LNR between Asian countries and non-Asia countries were also noteworthy. The cut-off values of LNR varied from 0.15 to 0.7 in different studies. Different prognostic values of LNR were shown based on different ranges of cut-off values. Significantly higher prognostic value was shown when the cut-off values of LNR ranging from 0.3 to 0.4 or greater than 0.5, although this possibly resulted from the small quantity of studies we could analyze in these 2 subgroups.

Some limitations of this meta-analysis should be mentioned. First, all included studies were retrospective studies which meant significant differences in patient characteristics, surgical plans, and other aspects might exist, leading to relatively low quality of data. Therefore, more prospective of large scale were expected to provide clinical data of ideal quality. Second, absence of data in tumor size, pathological stages, number of examined lymph node, number of metastasized lymph node and surgical methods limited further subgroup analysis. Third, the LNR cut-off values of some included articles were absent, which was likely to affect our exploration of the optimal range of LNR cut-off value in our subgroup analysis. Forth, the critical values of LNR in different studies were inconsistent. Although we grouped these studies by different ranges of critical value to analyze the cut-off value that best predicted recurrence, an exact optimal cut-off value was still left to be defined. Furthermore, moderate heterogeneity existed in this meta-analysis. However, we failed to seek single factor that caused heterogeneity through subgroup analysis and meta-regression with single covariate. We could only clarify that a combination of 5 factors including country, treatment, type of thyroid cancer, year and whether studies control factors in design or analysis influenced the heterogeneity of this analysis. Finally, we only included patients with papillary and medullary thyroid cancer, more studies discussing the prognostic role of LNR in other types of thyroid cancer were expected.

We should also point out the strength of our study. First, this was the first complete and formal meta-analysis to quantify the role of LNR in the prognosis of thyroid cancer. Second, 24 studies and a total of 15,698 patients were included in our meta-analysis, which allowed a relatively reliable statistical results. Thus, according to our analysis, the prognostic value of LNR was conspicuous.

In addition to the significant prognostic value, the potential of LNR to be included in the future thyroid cancer staging system may also be marked. Xu et al (39). proposed a new grading system combining the Ki67 index and LNR as a predictor of prognosis in medullary thyroid cancer. The prognostic performance of the new grading scheme was better than the Ki67, LNR and N staging alone. Lee et al (40). reported better predictive power of the new risk stratification systems combining metastasized lymph node factors including LNR, maximum diameter of metastatic focus and presence of extranodal extension with ATA risk stratification in N1 stage papillary thyroid cancer. In a word, more related studies are expected to establish a new risk stratification system and to assess the potential of LNR to be included in the system.

## 5 Conclusion

To conclude, higher LNR was correlated to poorer disease free survival in thyroid cancer and this result didn’t change in every subgroup we set. This study suggested that LNR could be a potential prognostic indicator for thyroid cancer. More effort should be made to assess the potential of LNR to be included in the future risk stratification systems for thyroid cancer.

## Data availability statement

The original contributions presented in the study are included in the article/supplementary material. Further inquiries can be directed to the corresponding author.

## Author contributions

YH: Data curation, Formal analysis, Investigation, Methodology, Resources, Validation, Visualization, Writing – original draft, Writing – review & editing. ZW: Data curation, Writing – review & editing. LD: Writing – review & editing. LZ: Writing – review & editing. LX: Funding acquisition, Resources, Supervision, Writing – review & editing.
